# The Influence of the Use of Different Polysaccharide Coatings on the Stability of Phenolic Compounds and Antioxidant Capacity of Chokeberry Hydrogel Microcapsules Obtained by Indirect Extrusion

**DOI:** 10.3390/foods12030515

**Published:** 2023-01-23

**Authors:** Marcelina Stach, Joanna Kolniak-Ostek

**Affiliations:** Department of Fruit, Vegetable and Plant Nutraceutical Technology, Wrocław University of Environmental and Life Sciences, 37 Chelmonskiego Street, 51-630 Wroclaw, Poland

**Keywords:** *Aronia melanocarpa*, microencapsulation, indirect extrusion, hydrogel beads, phenolic compounds, antioxidant capacity, bioactive compounds

## Abstract

The aim of the study was to evaluate the effect of the use of different polysaccharides and their mixtures on the structure of chokeberry hydrogel microcapsules and the stability of polyphenolic compounds and antioxidant capacity during one month of refrigerated storage. As a coating material, alginate and its mixtures with pectin, carrageenan, and chitosan were used, while chokeberry juice and preparation of polyphenolic compounds were used as a core. In non-stored capsules, the addition of carrageenan, pectin, and chitosan to alginate increased the content of total polyphenolic compounds and antioxidant capacity. When compared to non-encapsulated juice, the capsules had a greater decrease in antioxidant capacity during storage. The coating variant composed of alginate and carrageenan was found to be the most beneficial for the preservation of the capsules’ phenolic compounds. The findings revealed that proper polysaccharide coating selection is critical for the proper course of the microencapsulation process, the polyphenolic content of chokeberry capsules, and their antioxidant properties.

## 1. Introduction

The impact of dietary habits on human health and the occurrence of chronic non-communicable diseases (NCDs) has been widely investigated in recent years [1]. It has been proven that factors such as frequent consumption of fruit, vegetables, and their preserves and lower consumption of processed foods and trans fats have a positive effect on the incidence of NCDs [2]. Fruits are a rich source of bioactive compounds, such as polyphenols, with a wide range of diverse biological properties. Due to their antioxidant and anti-inflammatory activity, phenolic compounds can prevent NCDs, such as cancers, cardiovascular and gastrointestinal disorders, and type II diabetes [3,4,5,6].

Chokeberry fruit was chosen as a polyphenol source in this study. Black chokeberry (*Aronia melanocarpa*) (Michx. Elliott), a Rosaceae family shrub, is native to the eastern coast of the North American continent, where it grows wild. It is a rich source of polyphenols, including anthocyanins, polymeric procyanidins, monomeric flavan-3-ols, phenolic acids, and flavonols [3]. According to the literature [4], chokeberries have health-promoting beneficial effects on a variety of metabolic and immunological diseases, particularly those associated with oxidative stress. According to Sidor et al. [5], frequent consumption of fresh and processed chokeberry fruits prevents cardiovascular disease, controls blood pressure, lowers the negative effects of anticancer medications, alleviates irradiation effects, and protects the skin from damaging UV radiation. According to Fang et al. [6], in the case of chokeberry, this impact is mostly due to a high quantity of polyphenolic chemicals, particularly anthocyanins. Unfortunately, due to the low stability of polyphenols associated with high sensitivity to environmental factors, such as the presence of oxygen, light, or increased temperature, polyphenolic compounds lose their bioactive properties. An additional factor limiting their positive effect on the human body is their low stability in the gastrointestinal tract.

The limited stability of polyphenols limits their utility in the creation of functional foods and nutraceuticals. However, the microencapsulation process may be one way to reduce these undesirable losses. Microencapsulation involves building a protective polymer coating around the labile component to protect it from the impacts of external variables [7]. In addition to protecting active compounds such as dyes, fragrances, and antioxidants, microencapsulation allows for a controlled release of the core, delaying absorption in the gastrointestinal tract [8]. Mechanical (emulsification, spray-drying, fluidized-bed coating, centrifugal extrusion, spinning disks, pressure extrusion, and hot-melt extrusion) or chemical systems (ionotropic gelation, simple or complex coacervation, solvent evaporation, liposome entrapment, or cyclodextrin complexation) or a combination thereof can be used to produce capsules [9,10]. Depending on the obtained capsule size, they can be categorized as macro- (>1000 μm), micro- (1–1000 μm), and nanocapsules (<1 μm).

Extrusion using natural polymers is a technique that improves the stability of bioactive compounds, limits the use of high temperatures and organic solvents, and is additionally low-cost [11]. Sodium alginate obtained from brown seaweed has become the most popular polymer used in this technique due to its non-toxicity, low cost, and gentle gelling process in combination with cations such as Ca^2+^ via cross-linking the glucuronate carboxylate groups on the polymer backbone [12,13]. However, the beads thus formed have low stability and high porosity, which can lead to a burst effect [14].

Therefore, attempts are made to increase the barrier properties of alginate beads by using a mixture of alginate with complementary polymers [13,14]. Combinations of alginate with pectin, chitosan, or carrageenan have been examined for the gelation of bioactive compounds and drugs [14,15].

Carrageenan, a natural polysaccharide, is derived from red seaweeds or *Rhodophyta*. In the presence of calcium cations, it forms elastic, stable gels. It is widely phased out in pharmacies to improve drug release and formulation [16]. Chitosan is obtained from natural sources such as crustaceans and fungal cell walls. Due to its high compatibility with biomaterials, it can strengthen the structure of covering membranes, e.g., from alginate, by increasing their chemical and physical stability [17]. Pectin is one of the most used polysaccharides in food processing due to its gelling properties. Similar to alginate, it forms a polymer network in the presence of Ca^2+^ ions [18]. The mixture of alginate with other polysaccharides, such as pectin or carrageenan, in order to increase the stability and improve the mechanical properties of the capsules seems to be justified due to the similar gelling mechanism.

Due to the lack of research on microencapsulation of bioactive compounds derived from chokeberry using techniques other than spray drying, these studies focus on obtaining microcapsules of chokeberry juice and a phenolic preparation using the indirect extrusion method, with alginate, pectin, carrageenan and chitosan as coating materials. The influence of the applied polysaccharides on the content of polyphenolic compounds, antioxidant activity, and shape of the capsules was investigated. In addition, the stability of the capsules obtained after 30 days of storage was tested. It is assumed that the mixtures of coating polysaccharides used in the research will make it possible to increase the stability of the microcapsules during storage, which will preserve the high content of polyphenolic compounds and the antioxidant capacity of the tested beads and indicate their potential use in the production of food with enhanced health-promoting properties.

## 2. Materials and Methods

### 2.1. Materials, Reagents, and Standards

Coating polysaccharides: sodium alginate, low-molecular-weight chitosan, carrageenan, methanol, 2,2′-azinobis(3-ethylbenzothiazoline-6-sulfonic acid) (ABTS), 6-hydroxy-2,5,7,8-tetramethylchroman-2-carboxylic acid (Trolox), 2,4,6-tri(2-pyridyl)-s-triazine (TPTZ), formic acid, and calcium chloride were purchased from Sigma–Aldrich (Steinheim, Germany). Acetonitrile was purchased from Merck (Darmstadt, Germany). Low-methoxyl pectin was purchased from AGNEX (Białystok, Poland). (+)-Catechin, (−)-epicatechin, procyanidin B2, chlorogenic, neochlorogenic, coumaroylquinic and caffeic acid, quercetin-3-*O*-glucoside, quercetin-3-*O*-galactoside, quercetin-3-*O*-rutinoside, isorhamnetin-3-*O*-glucoside, eriodyctiol-glucuronide, cyanidin-3-*O*-galactoside, and cyanidin-3-*O*-glucoside were purchased from Extrasynthese (Lyon, France).

The experimental material consisted of chokeberry fruits of the cultivar Galicyjanka purchased at retail. The chokeberry juice was produced by pressing the chokeberry fruits on a hydraulic press (SSRE, Warsaw, Poland) (at a piston thrust of 15 tonnes of pressure for 2 min) and filtering through cotton wool. After this procedure, part of the material was transferred to a column containing Amberlite XAD-16 resin. The juice was washed with redistilled water (HYDROLAB HLP200, Gdańsk, Poland) to remove the sugars, organic acids, and high-molecular-weight compounds. Polyphenolic compounds were eluted with 80% ethanol (*v*/*v*). The resulting preparation of polyphenolic compounds was transferred to a vacuum evaporator (Hei-VAP Expert Control; Heidolph, Schwabach, Germany) to evaporate the ethanol and concentrate the solution.

### 2.2. Preparation of Hydrogel Beads

In order to obtain microcapsules, the procedure given in the BÜCHI B-390 Encapsulator manual has been followed [19]. Hydrogel beads were obtained with the indirect extrusion method. Encapsulation mixtures consisted of 3% CaCL_2_ (g/v) in chokeberry juice or the chokeberry polyphenolic preparation and 0.6% sodium alginate in water (g/v). In order to determine the influence of the applied polymer on the content of bioactive compounds, three additional mixtures were prepared in which alginate was mixed in equal proportions (1:1, g/g) with (1) low methylated pectin, (2) low-molecular chitosan, (3) carrageenan. The Encapsulator B-390 (BÜCHI Labortechnik AG, Flawil, Switzerland) was used to make hydrogel beads under the following conditions: 150 μm vibrating nozzle, 200 mbar pressure, frequency 400 Hz, and electrode 1000 V. The temperature of solidification was 30 °C. The complexation time was 10 min. After this time, the obtained hydrogel beads were rinsed with distilled water and used for further analysis.

### 2.3. Extraction Procedure

In order to prepare the stock solution, the method described by Kopjar et al. [20], with slight modifications, was used. 1 g of hydrogel beads was dissolved in 5 mL of 80% methanol acidified with HCl. The samples were placed in an ultrasonic bath (Sonic 6D, Polsonic, Warsaw, Poland) for 15 min at 20 °C and then placed in a refrigerator for 24 h. After this time, extracts were centrifuged at 3600 rpm for 20 min, and the supernatants were filtered through a hydrophilic PTFE 0.20 µm membrane (Millex Samplicity Filter, Merck, Darmstadt, Germany) and used for analysis.

### 2.4. Identification and Quantification of Phenolic Compounds

In order to identify and quantify polyphenols, an ACQUITY UPLC G2 Q-TOF system with a PDA detector (Waters Corporation, Milford, MA, USA) was used. Polyphenols were separated at 30 °C using a UPLC BEH C18 column (1.7 μm, 2.1 mm x 100 mm, Waters Corporation, Milford, MA, USA) for 15 min [21]. The mobile phase consisted of 4.5% formic acid (solvent A) and 100% acetonitrile (solvent B). Anthocyanins were measured at 520 nm, phenolic acids at 320 nm, and flavonols at 360 nm and 280 nm (flavan-3-ols and flavanones). Calibration curves were determined experimentally for (+)-catechin, (−)-epicatechin, procyanidin B2, chlorogenic acid, neochlorogenic acid, coumaroylquinic acid, caffeic acid, quercetin-3-*O*-glucoside, quercetin-3-*O*-galactoside, quercetin-3-*O*-rutinoside, eriodyctiol-glucuronide, isorhamnetin-3-*O*-glucoside, cyanidin-3-*O*-galactoside, and cyanidin-3-*O*-glucoside at concentrations ranging from 0.05 to 5 mg/mL (precision of calibration curves not < r2 = 0.9998). Cyanidin derivatives were expressed as cyanidin-3-*O*-galactoside, isorhamnetin derivatives were expressed as isorhamnetin 3-*O*-glucoside, and quercetin derivatives were expressed as quercetin-3-*O*-glucoside. All experiments were performed in triplicate. The results are expressed as milligrams per 100 g; the values represent the mean ± standard deviation.

### 2.5. Determination of Antioxidant Activity by DPPH, ABTS, and FRAP Assays

The DPPH, ABTS, and FRAP assays were performed as previously described by Yen and Chen [22], Re et al. [23], and Benzie and Strain [24], respectively. Briefly, in all analyses, 10 µL of the diluted samples (×10 dilution was used) and 200 µL of the DPPH, ABTS, and FRAP solutions were dispensed to the wells. DPPH was monitored at λ = 517 nm at 10 min; ABTS activity was measured after 6 min, and the absorbance at λ = 734 nm. The reduction forced of Fe^2+^ ions was measured at a wavelength of λ = 593 nm after 6 min. All measurements were performed on a Synergy H1 microplate reader (BioTek, Winooski, VT, USA). The results are expressed as mmol of Trolox equivalents (TE) per 100 g of the extract (mmol TE/100 g); the values represent the mean ± standard deviation.

### 2.6. Optical Microscopy Analysis

Optical analysis was performed according to an Axiolab 5 microscope manual [25]. An Axiolab 5 microscope (Zeiss, Jena, Germany) equipped with an Axiocam 208 microscope camera was used to image and measure the microspheres. For the analysis, the sample was examined at a magnification of 100.

### 2.7. Statistical Analysis

The results are presented as the means of three replications. Statistical analyses were performed with Statistica version 13.1 (StatSoft, Tulsa, OK, USA) based on Duncan’s test. The differences were considered to be significant at *p* ≤ 0.05.

## 3. Results

### 3.1. Analysis of Polyphenolic Compounds

Table 1 shows the profile and polyphenolic concentration (mg/100 g) in the chokeberry juice and polyphenolic preparation. The analysis showed the presence of polyphenolic compounds typical of chokeberry, belonging to anthocyanins, phenolic acids, flavan-3-ols, flavonols, and flavanones. The content of polyphenolic compounds in the juice and chokeberry preparation differed significantly. The purified preparation contained over five times more polyphenols than the juice. Comparable content of polyphenolic compounds in chokeberry juice (19.51 g/L) was obtained in the research by Lachowicz, Oszmiański, Kolniak-Ostek, and Stokłosa [21]. In the present study, the dominant fraction of polyphenolic compounds were anthocyanins, constituting over 42% (930.84 mg/100 g) of polyphenolic compounds in juice and over 48% (4801.15 mg/100 g) in the preparation. The second fraction in terms of content was phenolic acids, constituting 41% (896.63 mg/100 g) of polyphenolic compounds in the juice and 29% (288.41 mg/100 g) in the preparation. This group consisted of neochlorogenic, chlorogenic, and 3-*O*-p-coumaroylquinic acid. Chlorogenic acid and neochlorogenic acid were the major phenolic acids in chokeberry juice and preparation, constituting approximately 77% and 20%, respectively, in juice and 55% and 39%, respectively, in the preparation. The obtained results are consistent with the data presented by Lachowicz et al. [21].

In the present study, eight anthocyanins were identified, the dominant one being cyanidin-3-*O*-galactoside, accounting for over 55% (520.45 mg/100 g) of anthocyanins, followed by cyanidin-3-*O*-arabinoside, with 294.03 mg/100 g (over 31%). The results of our research agree with the observations of Płatosz et al. [26], who observed the same anthocyanin profile. In the case of chokeberry juice, the third most concentrated group was flavan-3-ols. Their content was 11%. In this group, (-)-epicatechin was the dominant compound, constituting almost 82% of the content. Similar results were obtained by Lachowicz, Oszmiański, and Kolniak-Ostek [27]. In their research, epicatechin constituted over 57% of compounds from the flavan-3-ol group. The flavonols were the third largest constituent in the polyphenolic preparation, accounting for over 11% of the total polyphenolic compounds. Eight compounds have been identified in this group: seven quercetin derivatives and one isorhamnetin derivative. The dominant compound was quercetin-glucoside, accounting for over 26% (309.71 mg/100 g) of flavonols (Table 1). Oszmiański and Lachowicz [28] obtained similar results in their research. The smallest group of polyphenolic compounds in both samples was flavanones (Table 1). Their total content was only 1.64% in the juice and 2.37% in the polyphenol preparation.

Table 2 shows the content of polyphenolic groups in non-capsulated juice and chokeberry preparation and hydrogel microcapsules before and after one month of storage. In the chokeberry preparation, the total content of polyphenolic compounds before storage was 4.5 times higher than in chokeberry juice. Hydrogel beads obtained from chokeberry preparation were 2.2 to 2.4 times higher in polyphenolic compounds than capsules obtained from chokeberry juice. In the group of unstored hydrogel beads, the highest concentration of polyphenolic compounds was determined in the variant with alginate and carrageenan (Alg + Carr): 584.64 mg/100 g in beads with chokeberry juice and 1102.40 mg/100 g in beads with chokeberry preparation. The use of chitosan and pectin as additives to alginate resulted in obtaining similar, average results of the content of polyphenols, with a slight advantage of chitosan. For chokeberry juice, the content of polyphenols in microcapsules with chitosan (Alg + Chit) was higher by 11%, while in the case of chokeberry juice, it was 3% higher than in capsules with pectin (Alg + Pect). The lowest content of polyphenols was determined in capsules obtained with alginate without the addition of other polymers: 416.76 mg/100 g and 988.84 mg/100 g for the juice and preparation, respectively.

The dominant group of polyphenolic compounds in the tested microcapsules was anthocyanins, followed by phenolic acids. In the case of capsules obtained from chokeberry juice, the content of anthocyanins ranged from 44.3% for the alginate variant to 49.8% in Alg + Chit. For the chokeberry preparation, these values ranged from 42.2% in Alg + Carr to 44.2% in Alg + Pect. The second largest group of polyphenolic compounds in all variants of microcapsules was phenolic acids. Their content ranged from 36.9% in Alg + Carr to 42.3% in mono-component capsules for chokeberry juice and from 34.0% in Alg + Chit to 40.0% in mono-component capsules for the chokeberry preparation. The flavanones were the group present in the smallest amount. Their content in capsules made of chokeberry juice ranged from 1.1% in alginate, Alg + Chit, and Alg + Carr to 1.2% in Alg + Pect, while in capsules obtained from the preparation of polyphenols, these values ranged from 1.7% in alginate to 3.2% in Alg + Chit. Capsules obtained from the preparation of chokeberry and a mixture of alginate and carrageenan (Alg + Carr) were characterized by a different composition. In their case, the smallest group of polyphenol compounds was flavonols, the content of which was 3.7% (Table 2).

The storage of non-capsulated samples and microcapsules for 1 month in refrigeration resulted in a reduction in the content of polyphenolic compounds. A decrease was noted both in the total number of polyphenols and in individual groups of compounds. The content of total number of polyphenols in the non-encapsulated juice decreased by 51% and in the polyphenolic preparation by 52%. The highest content of total polyphenols after refrigerated storage, both in microcapsules obtained from juice and from the preparation, was determined in the Alg + Carr variant (345.19 mg/100 g and 939.41 mg/100 g, respectively). The reduction in the content of polyphenolic compounds in relation to unstored capsules was 37.1% for the juice capsules and 23.9% for the capsules from the polyphenol preparation. The second largest amount of total polyphenols after storage was observed for microcapsules from the Alg + Pect variant. The content of total polyphenols in hydrogel beads made from juice was 295.45 mg/100 g, while in capsules from a polyphenol preparation, the content of polyphenols was 652.91 mg/100 g. Polyphenols were best preserved in microcapsules made of a mixture of alginate and pectin and alginate and carrageen. In capsules made of juice, the content of polyphenols was reduced to 67% and 63%, respectively, while in capsules of the chokeberry preparation, the content of polyphenols after storage was 66% and 76%, respectively. The lowest concentration of total polyphenolic compounds after storage was determined in microcapsules coated with Alg + Chit: 205.84 mg/100 g in hydrocapsules from juice and 508.26 mg/100 g in capsules from the polyphenol preparation. At the same time, the reduction in the content of total polyphenols in these variants was the greatest and was 58.5% for the juice and 53.9% for the polyphenol preparation (Table 2). The obtained results clearly show the positive effect of encapsulation on the stability of chokeberry polyphenolic compounds and the need to use an appropriate encapsulating mixture in order to reduce the loss of bioactive compounds.

The content of individual groups of polyphenolic compounds in the samples not subjected to encapsulation decreased during one-month storage. The largest decrease in content, amounting to 73% for the juice and 71% for the preparation, was observed in the group of flavonols. Compounds from the group of flavanones were also significantly reduced. Their content after storage in the juice decreased by 71% and in the preparation by 62%. In the groups of anthocyanins and phenolic acids, the content reduction was 51% and 55% for the juice and 52% and 56% for the preparation, respectively. Flavan-3-ols were characterized by the highest storage stability. Their content after 1 month decreased by 21% both in the juice and in the chokeberry preparation. During storage, different effects of the applied protective coatings on individual groups of polyphenolic compounds were observed. In the case of anthocyanins, the application of Alg + Pect turned out to be the most effective: 76.1% of compounds were retained in capsules made of chokeberry juice, and 79.3% in capsules made of polyphenol preparation. The least effective coating for these compounds was a mixture of alginate with chitosan: after storing the juice capsules, only 46.2% of anthocyanins were retained, while in capsules made of the polyphenol preparation, it was 62.5%. In the case of the remaining groups of polyphenolic compounds, the results are heterogeneous. It can be noted, however, that in all cases, the use of a mixture of alginate with chitosan (Ag + Chit) was the least effective in protecting polyphenolic compounds against oxidation. In the case of capsules made of chokeberry juice, the residue of individual groups of polyphenols after storage ranged from 24.3% for flavan-3-ols to 42.3% for polyphenolic acids, while in capsules made from a polyphenolic preparation, it was from 46.1% for the group of flavanones to only 19.5% for flavan-3-ols (Table 2).

The protective effect of microencapsulation on polyphenolic compounds during storage is consistent with previously published data. Lin et al. [29], in studies on astaxanthin encapsulated with various alginate solutions, obtained significantly higher polyphenol stability during 21 days of storage compared to the non-encapsulated solution. Phenolic compounds are extremely unstable and prone to breakdown during storage. The speed of degradation is affected by a number of factors, including time, light, temperature, and the type of processing utilized in our study addition of different polysaccharides to the coating material affected the encapsulation of total and individual polyphenols. This may be related to the different mechanisms and rate of complexation of the polysaccharides with calcium ions. According to Zam, Bashour, Abdelwahed & Khayata [30], when calcium and alginate came into contact, the gel formed directly at the interface, but the homogeneity of the matrix was dependent on calcium diffusion. It has been found that exposing the alginate to calcium ions for an extended period of time causes additional calcium to diffuse into the gel network and bind to the alginate. Longer durations, on the other hand, may result in the release of polyphenols into the matrix or the shifting of the confined calcium ions by alginate. The diffusion of calcium into the gel network may be affected by the changes in the composition of polysaccharides. The polarity of chemicals also influences their encapsulation inside alginate beads. Poor encapsulation occurs for more hydrophobic polyphenols due to non-incorporation into the encapsulation mixture, which typically consists of hydrophilic components such as alginate, and hence their encapsulation remains a challenge [31]. Other parameters influencing interactions with polysaccharides via covalent and hydrogen bonding included the quantity and position of phenolics hydroxyl groups [13]. Because food is a complex matrix, phenolics can interact with one another or with other food components, changing their properties. Sugars and hydroxyl groups at different locations in the B ring of flavonols changed their interactions with polysaccharides [32].

The obtained results clearly indicate the positive effect of microencapsulation on polyphenolic compounds. It was found that the best mixture to preserve polyphenols was the Alg + Carr variant and in the case of anthocyanins, the Alg + Pect coating (Table 2). Further research is required on the composition and conditions of the production process of polysaccharide beads, which will allow for greater stability of polyphenolic compounds, especially from the group of anthocyanins, during storage.

### 3.2. Analysis of Antioxidant Capacity

Table 3 presents the values of the antioxidant capacity of the chokeberry juice and preparation, as well as the microcapsules obtained from them, before and after 1 month of refrigerated storage. The table also includes the values of the correlation coefficient between the DPPH, ABTS, and FRAP antioxidant capacities and the individual groups of chokeberry polyphenols.

The chokeberry preparation had greater DPPH, ABTS, and FRAP antioxidant capacity values compared to chokeberry juice (Table 3). The highest antioxidant activity of the phenolic preparation was measured by the DPPH method (15.23 mmol TE/100 g). Lower values were obtained for ABTS and FRAP methods: 14.33 mmol TE/100 g and 14.19 mmol TE/100 g, respectively. Chokeberry juice was characterized by 1.7 to 3.2 times lower antioxidant capacity for DPPH and FRAP methods, respectively. Chokeberry juice was characterized by 2 to 4 times lower antioxidant capacity for DPPH and FRAP methods, respectively. Our results are slightly lower than those obtained by Oszmiański and Lachowicz [28]. In juices produced from chokeberry without crushing before pressing, the ABTS and FRAP values were respectively 20.11 and 9.81 mmol Tx/100 g dm, while in powders obtained from uncrushed fruits, these values were 81.62 and 53.78 mmol Tx/100 g dm.

Encapsulation significantly influenced the value of the antioxidant capacity. The capsules obtained using a mixture of alginate and carrageenan were characterized by the highest antioxidant capacity. In the hydrogel pearls obtained from chokeberry juice, the values of DPPH, ABTS, and FRAP were 0.95, 0.70, and 0.72 mmol TE/100 g, respectively, while in the capsules of the polyphenol preparation, the values were 1.41, 1.39 and 1.89 mmol TE/100 g. The lowest ability to reduce free radicals was observed in the case of the mono-component capsule. For chokeberry juices, the values of DPPH, ABTS, and FRAP were 0.9, 0.66, and 0.65 mmol TE/100 g, respectively, while for the chokeberry preparation, the values were 1.25, 1.19, and 1.34 mmol TE/100 g.

The storage significantly influenced the value of the antioxidant capacity. In all tested variants, there was a decrease in the ability to reduce free radicals of the tested capsules. In the group of capsules made of chokeberry juice, the greatest protective properties were observed in the mixture of alginate and carrageen (Alg + Carr). After one month of storage, the antioxidant capacity of DPPH, ABTS, and FRAP was maintained at 98%, 97%, and 99%, respectively. For comparison, at the same time of storage, the value of the antioxidant capacity of the non-encapsulated juice decreased to 76%, 81%, and 86%, respectively. The worst results were obtained for capsules prepared with a mixture of alginate and chitosan (Alg + Chit). In this case, the antioxidant capacity values dropped to 69% for DPPH, 58% for ABTS, and 21% for FRAP (Table 3).

In the group of capsules obtained from the chokeberry preparation, greater changes in the antioxidant capacity were observed during storage. In the case of the non-encapsulated formulation, the antioxidant capacity values were reduced to 56%, 55%, and 58% for DPPH, ABTS, and FRAP, respectively. Again, the value of the antioxidant capacity was preserved to the greatest extent when a mixture of alginate and carrageenan (Alg + Carr) was used. In this case, the antioxidant capacity after storage was 95%, 91%, and 90% of the original value, respectively. Chitosan once again proved to be the least effective coating. In Alg + Chit capsules, the value of the antioxidant capacity decreased by 52% for DPPH, 69% for ABTS, and 81% for FRAP.

The values of antioxidant capacity were positively correlated with the content of polyphenols. The correlation coefficient between the sum of polyphenols and DPPH capacity was 0.85, while for ABTS and FRAP, it was 0.95 and 0.96, respectively (Table 3). The individual groups of polyphenolic compounds also had a great influence on the antioxidant capacity. The highest correlation coefficient between the three tests of antioxidant capacity was determined for compounds from the group of anthocyanins and phenolic acids. The smallest degree of radical scavenging was influenced by the flavanones. Polyphenols have several hydroxyl groups and can neutralize free radicals in the body and also convert free radicals into stable materials, effectively stopping free radical chain reactions and delaying or blocking many diseases [32,33]. Several studies have been conducted over the last two decades to investigate the relationship between the structure and antioxidant activity of diverse phenolic compounds [34,35]. Numerous reports have detailed the structural aspects, mechanism, and molecular properties that determine flavonoids’ antioxidant activity; among these, several studies have used theoretical calculations such as density functional theory (DFT) [35]. Polymerization of monomers such as (-)epicatechin and (+)catechin results in the formation of flavanols. They can be monomeric, oligomeric, or polymeric to a high degree. Flavanols have excellent antioxidant capabilities due to their structure [36,37]. Proanthocyanidins reduce oxidative stress by regulating oxidative stress signaling pathways, limiting ROS generation, oxidative stress damage, and apoptosis-related pathways, and inhibiting the stress-activated MAPK pathway [38]. Anthocyanins have high antioxidant activity, which is determined by the location and number of hydroxyl groups, the degree of glycation, and the presence of electron donors in their structure [3].

### 3.3. Microscopic Analysis

**Figure 1:**
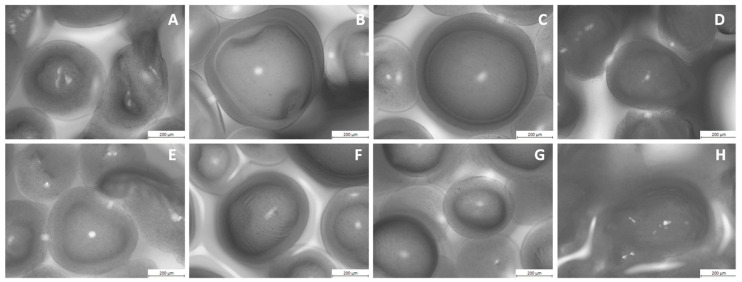
Microscopic images of microcapsules obtained from chokeberry juice and preparation with different coating materials: (**A**) chokeberry juice, coating: alginate; (**B**) chokeberry juice, coating: alginate + pectin; (**C**) chokeberry juice, coating: alginate + carrageenan; (**D**) chokeberry juice, coating: alginate + chitosan; (**E**) chokeberry preparation, coating: alginate; (**F**) chokeberry preparation, coating: alginate + pectin; (**G**) chokeberry preparation, coating: alginate + carrageenan; (**H**) chokeberry preparation, coating: alginate + chitosan.

Figure 1 shows a microscopic image of capsules obtained from the chokeberry juice and polyphenolic preparation using different coatings.

The analysis showed that the hydrogel beads differ in shape and structure. The most regular capsules were obtained in the example of the Alg + Carr mixture (Figure 1C,G). The capsules had a round shape with a clear, fluid center and a uniform cover. Capsules obtained from a mixture of alginate and pectin (Figure 1B,F) also had a clearly separated core and cover, while their shape was less regular. Their structure shows the thickening and folding of the capsule walls. The mono-component microcapsules (Figure 1A,E) were irregular in shape and size. Within the microscopic image, one can also see gelled alginate, which did not form capsules with the juice and chokeberry preparation. The last regular structure was found in capsules obtained from the mixture of Alg + Chit (Figure 1D,H). In the case of the variant with the chokeberry preparation, it was not possible to obtain hydrogel beads but only a gelled structure in which the polyphenol preparation was enclosed. In the variant of chokeberry juice, the walls of the capsules can be seen, although they are characterized by high irregularity.

## 4. Conclusions

The conducted research has shown the possibility of producing hydrogel microcapsules from chokeberry juice and preparation using the indirect extrusion method and various coating polymers. It was found that a mixture of alginate and carrageenan, or alginate and pectin, is an effective protective coating, positively affecting the stability of chokeberry polyphenolic compounds during storage. Chokeberry hydrogel microcapsules are a good source of polyphenolic compounds with antioxidant properties. Further research is needed on the composition of the shell mixtures and on the conditions of indirect encapsulation in order to maintain high antioxidant activity during storage. Stable capsules can be used in the production of functional food with designed pro-health properties. They can also be used to enrich traditional foods such as fruit juices, mousses, and yogurts with bioactive substances. Chokeberry hydrogel beads also have potential applications in the cosmetic and pharmaceutical industries.

## Figures and Tables

**Table 1:** Profile and quantity (mg/100 g of product) of phenolic compounds in chokeberry juice and phenolic preparation.

RT (min)	UV _λmax_ (nm)	[M − H]^−^/[M + H]^+^ (*m/z*)	MS/MS (*m/z*)	Tentative Identification	Chokeberry Juice	Chokeberry Preparation
Anthocyanins						
3.28	242, 521	737.1551	575.1198/287.9778	Cyanidin-3-hexoside-(epi)catechin ^2^	23.05 ± 0.01 ^e^	160.44 ± 3.00 *^e^*
3.81	283, 524	707.1428	329.0815/287.0707	Cyanidin-3-pentoside-(epi)catechin ^2^	23.15 ± 0.01 ^e^	198.40 ± 3.99 *^d^*
4.04	282, 520	1025.209	575.1270/287.0711	Cyanidin-3-hexoside-(epi)catechin-(epi)catechin ^2^	27.42 ± 0.01 ^d^	230.22 ± 8.01 *^c^*
4.39	280, 520	449.1134	287.0675	Cyanidin-3-*O*-galactoside ^1^	520.45 ± 1.02 ^a^	2759.33 ± 23.19 *^a^*
4.86	279, 515	449.112	287.0672	Cyanidin-3-*O*-glucoside ^1^	39.59 ± 0.01 ^c^	250.91 ± 2.15 *^c^*
4.88	279, 514	419.1054	287.0676	Cyanidin-3-*O*-arabinoside ^2^	294.03 ± 1.05 ^b^	1015.24 ± 21.02 *^b^*
5.51	279, 515	419.1037	287.0732	Cyanidin-3-*O*-xyloside ^2^	1.17 ± 0.00 ^g^	87.24 ± 2.79 *^g^*
6.31	280, 515	287.0709		Cyanidin ^2^	1.98 ± 0.00 ^f^	99.37 ± 1.78 *^f^*
Phenolic acids						
3.26	322	353.097	191.0697/179.0483	Neochlorogenic acid ^1^	180.22 ± 1.21 ^b^	1128.43 ± 25.87 *^b^*
4.01	311	337.9106	191.0766	3-*O*-*p*-Coumaroylquinic acid ^1^	22.37 ± 0.20 ^c^	150.53 ± 5.15 *^c^*
4.37	322	353.0978	191.0699	Chlorogenic acid ^1^	694.04 ± 2.41 ^a^	1587.45 ± 43.44 *^a^*
Flavan-3-ols						
3.78	285	289.0684		(+)-Catechin ^1^	10.69 ± 0.20 ^c^	87.92 ± 3.98 *^c^*
5.34	277	289.0881		(-)-Epicatechin ^1^	199.66 ± 1.56 ^a^	570.43 ± 14.36 *^a^*
6.74	280	577.1315	289.0522	Procyanidin B2 ^1^	33.83 ± 0.12 ^b^	180.35 ± 5.12 *^b^*
Flavonols						
6.23	255, 353	625.1376	301.0378	Quercetin-di-hexoside ^2^	0.26 ± 0.00 ^g^	38.35 ± 2.77 *^f^*
6.30	260, 353	625.1610	301.0514	Quercetin-di-hexoside ^2^	0.64 ± 0.00 ^g^	40.54 ± 3.01 *^f^*
6.62	255, 353	595.1298	431.1937/301.0433	Quercetin-3-*O*-vicianoside ^2^	6.36 ± 0.02 ^d^	180.66 ± 9.19 *^c^*
6.84	230, 323	609.2298	301.0441	Quercetin-3-*O*-robinobioside ^2^	17.81 ± 0.01 ^b^	215.15 ± 8.22 *^b^*
7.00	255, 320	609.1415	301.0403	Quercetin-3-*O*-rutinoside ^1^	9.91 ± 0.01 ^c^	176.36 ± 8.98 *^c^*
7.12	255, 352	463.0899	301.0455	Quercetin-3-*O*-galactoside ^1^	3.79 ± 0.01 ^e^	140.22 ± 6.76 *^d^*
7.22	255, 352	463.0901	301.0428	Quercetin-3-*O*-glucoside ^1^	39.89 ± 0.02 ^a^	309.71 ± 10.47 *^a^*
7.79	265, 345	623.1624	463.0815/315.7207	Isorhamnetin rhamnosyl-hexoside ^2^	1.12 ± 0.00 ^f^	63.72 ± 3.12 *^e^*
Flavanones						
7.4	287	281.0098	463.1537	Eriodictyol glucuronide ^1^	35.99 ± 0.22 ^a^	234.97 ± 9.18 *^a^*
				SUM	2187.42 ± 5.48 ^B^	9905.94 ± 89.97 ^A^

^1^ Identification confirmed by commercial standards. ^2^ Identification by comparison of MS data with the literature; identification is tentative. Values (mean of three replications) ± standard deviation followed by different letters (a–f) and (*a–f*) within the same group of compounds are different (*p* ≤ 0.05) according to Duncan’s test. Values (sum of compounds) followed by different letters (A,B) are different (*p* ≤ 0.05) according to Duncan’s test.

**Table 2:** Quantity (mg/100 g of product) of phenolic compounds in microcapsules made with chokeberry juice and chokeberry preparation before and after 1 month of storage at 4 °C.

		Total Anthocyanins		Total Phenolic acids		Total Flavan-3-ols		Total Flavonols		Total Flavanones		Total Phenolics	
		Not Stored	1 Month	Not Stored	1 Month	Not Stored	1 Month	Not Stored	1 Month	Not Stored	1 Month	Not Stored	1 Month
			of Storage		of Storage		of Storage		of Storage		of Storage		of Storage
	non-caps	930.84 ± 21.23 ^b^	450.99 ± 11.48 ^b^	896.63 ± 17.71 ^b^	406.11 ± 12.98 ^b^	244.18 ± 9.47 ^b^	193.00 ± 9.82 ^b^	79.78 ± 2.01 ^b^	21.57 ± 0.32 ^c^	35.99 ± 0.32 ^b^	10.28 ± 0.19 ^cd^	2187.42 ± 5.48 ^b^	1081.95 ± 17.47 ^b^
Chokeberry	Alginate	184.73 ± 5.43 ^h^	101.99 ± 8.39 ^h^	176.40 ± 5.37 ^g^	120.88 ± 4.81 ^f^	40.61 ± 1.12 ^f^	27.19 ± 0.41 ^f^	10.64 ± 0.12 ^e^	6.40 ± 0.12 ^e^	4.38 ± 0.14 ^f^	2.60 ± 0.12 ^f^	416.76 ± 8.93 ^j^	259.06 ± 8.84 ^g^
Juice	Alg + Pect	201.15 ± 7.17 ^g^	153.12 ± 10.12 ^g^	180.22 ± 4.99 ^fg^	100.23 ± 5.25 ^g^	43.59 ± 2.43 ^f^	32.61 ± 0.36 ^f^	11.01 ± 0.35 ^e^	6.50 ± 0.22 ^e^	5.15 ± 0.21 ^e^	2.99 ± 0.15 ^f^	441.12 ± 9.54 ^i^	295.45 ± 7.32 ^f^
	Alg + Chit	215.32 ± 8.55 ^g^	99.52 ± 5.67 ^h^	201.17 ± 5.68 ^f^	85.15 ± 1.34 ^h^	62.18 ± 2.99 ^e^	15.12 ± 0.29 ^g^	12.32 ± 0.68 ^e^	4.20 ± 0.14 ^f^	5.25 ± 0.42 ^e^	1.85 ± 0.04 ^f^	496.24 ± 9.28 ^h^	205.84 ± 9.47 ^h^
	Alg + Carr	259.59 ± 10.25 ^f^	161.92 ± 9.58 ^g^	202.34 ± 5.13 ^f^	123.12 ± 4.32 ^f^	65.65 ± 1.18 ^e^	45.38 ± 0.61 ^e^	15.05 ± 0.47 ^e^	10.21 ± 0.98 ^d^	6.01 ± 0.55 ^e^	4.56 ± 0.09 ^e^	548.64 ± 10.42 ^g^	345.19 ± 6.16 ^e^
	non-caps	4801.15 ± 48.12 ^a^	2304.58 ± 33.21 ^a^	2866.41 ± 29.19 ^a^	1289.88 ± 17.36 ^a^	838.70 ± 17.71 ^a^	662.57 ± 13.72 ^a^	1164.71 ± 15.62 ^a^	349.47 ± 11.27 ^a^	234.97 ± 10.10 ^a^	87.98 ± 9.87 ^a^	9905.94 ± 89.97 ^a^	4694.48 ± 32.91 ^a^
Chokeberry	Alginate	429.04 ± 9.88 ^e^	278.22 ± 4.93 ^f^	395.06 ± 7.59 ^d^	161.95 ± 6.63 ^d^	117.04 ± 2.73 ^d^	56.48 ± 0.94 ^e^	30.82 ± 1.12 ^d^	18.08 ± 0.76 ^c^	16.90 ± 0.76 ^d^	8.85 ± 1.01 ^d^	988.84 ± 22.61 ^f^	523.58 ± 19.93 ^d^
Preparation	Alg + Pect	472.78 ± 11.45 ^d^	340.42 ± 7.46 ^d^	404.08 ± 7.63 ^d^	179.75 ± 5.72 ^d^	128.72 ± 3.01 ^d^	88.26 ± 1.25 ^d^	40.19 ± 0.98 ^c^	26.53 ± 1.03 ^b^	24.90 ± 0.69 ^c^	17.97 ± 1.22 ^c^	1070.67 ± 24.86 ^e^	652.91 ± 18.89 ^c^
	Alg + Chit	484.61 ± 12.22 ^d^	303.12 ± 8.33 ^e^	374.64 ± 9.23 ^e^	140.69 ± 3.55 ^e^	168.61 ± 2.98 ^c^	32.93 ± 1.01 ^f^	38.98 ± 1.32 ^cd^	17.97 ± 1.00 ^c^	35.57 ± 1.48 ^b^	13.55 ± 0.98 ^c^	1102.40 ± 26.26 ^d^	508.26 ± 19.27 ^d^
	Alg + Carr	521.07 ± 11.89 ^c^	400.51 ± 7.15 ^c^	439.78 ± 8.18 ^c^	321.95 ± 7.64 ^c^	180.56 ± 3.15 ^c^	159.97 ± 2.04 ^c^	46.19 ± 1.02 ^c^	28.54 ± 0.68 ^b^	47.34 ± 1.42 ^b^	28.43 ± 1.13 ^b^	1234.94 ± 23.878 ^c^	939.41 ± 19.99 ^a^

Alg—alginate; Pect—pectin; Chit—chitosan; Carr—carrageenan; non-caps—not capsulated; values (mean of three replications) ± standard deviation followed by different letters (a–k), within the same column are different (*p* ≤ 0.05) according to Duncan’s test.

**Table 3:** Antioxidant capacity (mmol TE/100 g) of microcapsules made with chokeberry juice and chokeberry preparation before and after 1 month of storage at 4 °C and correlation coefficient.

			DPPH	ABTS	FRAP
Chokeberry juice	Not stored	Non-encapsulated juice	8.80 ± 0.09 ^b^	5.04 ± 0.21 ^c^	4.49 ± 0.53 ^c^
		Alginate	0.90 ± 0.02 ^g^	0.66 ± 0.01 ^i^	0.65 ± 0.01 ^h^
		Alg + pect	0.91 ± 0.02 ^g^	0.67 ± 0.01 ^i^	0.67 ± 0.02 ^h^
		Alg + chit	0.94 ± 0.03 ^g^	0.69 ± 0.01 ^i^	0.71 ± 0.02 ^h^
		Alg + carr	0.95 ± 0.03 ^g^	0.70 ± 0.02 ^i^	0.72 ± 0.02 ^h^
	1 month of storage	Non-encapsulated juice	8.67 ± 0.06 ^bc^	4.90 ± 0.17 ^d^	4.36 ± 0.43 ^c^
		Alginate	0.77 ± 0.02 ^h^	0.56 ± 0.01 ^j^	0.29 ± 0.00 ^i^
		Alg + pect	0.80 ± 0.02 ^h^	0.58 ± 0.01 ^j^	0.35 ± 0.00 ^i^
		Alg + chit	0.65 ± 0.02 ^i^	0.40 ± 0.00 ^k^	0.15 ± 0.00 ^j^
		Alg + carr	0.93 ± 0.03 ^g^	0.68 ± 0.02 ^i^	0.71 ± 0.03 ^h^
Chokeberry preparation	Not stored	Non-encapsulated preparation	15.23 ± 0.91 ^a^	14.33 ± 0.92 ^a^	14.19 ± 0.81 ^a^
		Alginate	1.25 ± 0.06 ^f^	1.19 ± 0.04 ^g^	1.34 ± 0.06 ^f^
		Alg + pect	1.32 ± 0.06 ^e^	1.20 ± 0.05 ^g^	1.4 ± 0.07 ^f^
		Alg + chit	1.35 ± 0.05 ^de^	1.31 ± 0.04 ^f^	1.76 ± 0.08 ^e^
		Alg + carr	1.41 ± 0.06 ^d^	1.39 ± 0.^06 e^	1.89 ± 0.08 ^d^
	1 month of storage	Non-encapsulated preparation	8.50 ± 0.76 ^c^	7.90 ± 0.69 ^b^	8.20 ± 0.66 ^b^
		Alginate	0.95 ± 0.07 ^g^	0.69 ± 0.01 ^i^	0.70 ± 0.01 ^h^
		Alg + pect	0.99 ± 0.07 ^g^	0.83 ± 0.02 ^hi^	0.78 ± 0.03 ^g^
		Alg + chit	0.65 ± 0.05 ^i^	0.40 ± 0.01 ^k^	0.34 ± 0.00 ^i^
		Alg + carr	1.34 ± 0.08 ^de^	1.27 ± 0.07 ^f^	1.70 ± 0.06 ^e^
Correlation coefficient		Sum of phenolic compounds	0.85	0.95	0.96
		Anthocyanins	0.87	0.96	0.97
		Phenolic acids	0.90	0.97	0.98
		Flavan-3-ols	0.88	0.96	0.97
		Flavonols	0.82	0.92	0.92
		Flavanones	0.79	0.91	0.93

Values (mean of three replications) ± standard deviation followed by different letters (a–m) within the same column are different (*p* ≤ 0.05) according to Duncan’s test; Alg—alginate; Pect—pectin; Chit—chitosan; Carr—carrageenan.

## Data Availability

Data will be made available on request.
